# TRANSLATING RESEARCH TO PRACTICE: USING CHANGE MODEL TO IMPROVE SUSTAINABILITY OF HEALTH ALERTS FOR CHRONIC ILLNESS

**DOI:** 10.1093/geroni/igz038.2907

**Published:** 2019-11-08

**Authors:** Kari R Lane, Marilyn J Rantz, Marjorie Skubic

**Affiliations:** University of Missouri, Columbia, Missouri, United States

## Abstract

Chronic illness is the primary reason for hospitalization and rehospitalization in the US today. Nearly 1/3 of older adults have 3 or more chronic illnesses. Chronic illnesses require significant self-management or management by nursing staff. This paper highlights the use of a change model to assist in sustaining nursing interventions in assisted living environments. We utilized embedded sensors measuring heart rate, respiratory rate, time in bed, restlessness in bed, and gait parameters to manage chronic illness. The embedded sensors use an algorithm to signify when a measure has changed for a resident, based on the past 2 weeks of data. Early health messages are emailed or texted to nursing staff. Nursing staff can use these messages as tools to further assess the resident’s condition. It was important to revisit the education, hold the staff accountable, phone in suggestions/reinforcement of what the alerts meant, and provide positive messages. This interdisciplinary study has been deployed in 6 assisted living settings (n=386) (facility-wide) in the midwest. We used a wait-list control group (n=482) of facilities awaiting sensor installation. Outcome variables included length of stay, hospitalizations, falls, and medication changes. Results included a decrease in all outcome variables length of stay 1.98 years longer (F=3.67; p=0.003); hospitalizations (F=2.15; p=0.048); falls (F=1.899; p=0.012); and medication changes (F=3.9; p=0.0008) when compared to the control group. We feel these results may benefit other clinicians in the future when implementing new protocols and practices.

